# Detection of COVID-19 from CT scan images: A spiking neural network-based approach

**DOI:** 10.1007/s00521-021-05910-1

**Published:** 2021-04-16

**Authors:** Avishek Garain, Arpan Basu, Fabio Giampaolo, Juan D. Velasquez, Ram Sarkar

**Affiliations:** 1 Department of Computer Science and Engineering, Jadavpur University, Kolkata, 700032 India; 2 Department of Mathematics and Applications “R. Caccioppoli”, University of Naples Federico II, Naples, NA Italy; 3 Department of Industrial Engineering, Faculty of Physical and Mathematical Sciences, Instituto Sistemas Complejos de Ingeniería (ISCI), University of Chile, Santiago, Chile

**Keywords:** COVID-19, CT scan, Deep learning, Medical image, Spiking neural network

## Abstract

The outbreak of a global pandemic called coronavirus has created unprecedented circumstances resulting into a large number of deaths and risk of community spreading throughout the world. Desperate times have called for desperate measures to detect the disease at an early stage via various medically proven methods like chest computed tomography (CT) scan, chest X-Ray, etc., in order to prevent the virus from spreading across the community. Developing deep learning models for analysing these kinds of radiological images is a well-known methodology in the domain of computer based medical image analysis. However, doing the same by mimicking the biological models and leveraging the newly developed neuromorphic computing chips might be more economical. These chips have been shown to be more powerful and are more efficient than conventional central and graphics processing units. Additionally, these chips facilitate the implementation of spiking neural networks (SNNs) in real-world scenarios. To this end, in this work, we have tried to simulate the SNNs using various deep learning libraries. We have applied them for the classification of chest CT scan images into COVID and non-COVID classes. Our approach has achieved very high F1 score of 0.99 for the potential-based model and outperforms many state-of-the-art models. The working code associated with our present work can be found here.

## Introduction

Coronavirus disease 2019 (COVID-19) is an infectious respiratory disease caused by severe acute respiratory syndrome coronavirus 2 (SARS-CoV-2). It is responsible for an ongoing global pandemic. It has caused a large number of deaths worldwide, with the count of affected people increasing day by day. It has been detrimental to both the economy and the society. Since no vaccine has been developed, preventive measures such as social distancing, quarantining, use of face-masks, etc., have been recommended. The management of the disease involves prompt identification of infected individuals via wide-scale testing and isolation of the infected population. The optimal mode of testing would be by real-time reverse transcription polymerase chain reaction (rRT-PCR). Chest X-Rays and CT images have also been used, though they are not recommended. However, in many cases, X-Ray and CT images can be beneficial to radiologists in an initial screening process. In this work, an approach for detecting COVID-19 in CT images is discussed.

With the advent of high computing power and deep learning algorithms for leveraging the same, deep convolutional neural networks (DCNNs) have brought a drastic upliftment in the performances achieved in the domain of computer vision. In many cases, such models perform way better than human vision in various object recognition tasks. Even in the presence of models with high levels of working efficiency and accuracy, the search for computational algorithms which are inspired by the functioning of brains is continuously growing and has been a centre of attraction for researchers from all over the world. Considering this domain of research, a plethora of architectures and computational models based on spiking neural networks (SNNs) have been proposed. Though DCNNs outperform SNNs in terms of recognition accuracy, the obvious question that arises is that, if performance is not the key then what is the reason for this new trend of increasing interest towards neurobiologically modelled SNNs. Previously the problem used to be the limitation in computational abilities; however, in the present time and immediate future, the need of the hour is environment friendly, power-efficient algorithms and computational devices. The human brain has been evolving since a millions of years. The resultant optimization from this evolution has made this fact possible that the human brain consumes approximately 20W power. This is equivalent to the power consumption of an average laptop. Understanding this efficiency of brain is beyond our current capabilities, but using computation based on spikes has already helped researchers for designing neuromorphic energy-efficient microchips [3, 4]. The era of Internet of Things (IoT) calls for evolution of on-device artificial intelligence (AI)-based models. Biologically inspired learning mechanisms such as spike-timing-dependent plasticity (STDP) [1, 7] can be unimaginably friendly to hardware and might be the perfect match for online on-chip training [31]. Apart from these reasons, the natural ability of SNNs of handling spatio-temporal patterns has inspired the researchers to try various methods for applying SNNs for various visual tasks. Using structured neural networks in a hierarchical manner is one of the most common approaches, but configuration of other hyperparameters like the count of layers, neuron models and information encoding needs a lot of experimentation. In the context of brain-inspired algorithms, STDP-based SNNs are the most biologically plausible. By making use of STDP, the network can successfully extract those visual features which are the most frequently occurring. However, after feature extraction, for the purpose of decision making, external classifiers such as support vector machines (SVMs) and radial basis functions (RBFs) or supervised variants of STDP, are usually required.

Chest CT scans have been a well-proven functionality for providing assistance to the detection of COVID-19. Methods have been proposed for classifying these CT scan images to diagnose patients for COVID-19. However, to the best of our knowledge, application of SNNs for the same has not been considered by any researchers. In this work, we have used a 3-layer DCSNN (deep convolutional spiking neural network) with a structure adopted from [15] for the binary classification of COVID-19 from CT scan images. First, the input image is convolved and processed with Gabor filters at various scales and orientations. Then, by means of an intensity-to-latency encoding [6], a wave of spikes is generated and propagated to the next layer. After propagating through a series of convolutions and pooling layers with neurons which are capable of firing at most once, the spike wave finally reaches the penultimate layer. From this layer, the features are extracted in order to be fed to an external classifier for the purpose of final decision-making for class assignment. For every image that is fed to the network, the neurons present in the final layer with either the earliest spike time or maximum potential contributes to the decision of the network, respectively.

The rest of the paper has been organized as follows: Sect. 2 provides a literature survey about the works done on this topic. Section 3 describes the dataset on which the proposed framework has been evaluated. The methodology followed in the present work is described in Sect. 4. This is followed by the results and concluding remarks in Sects. 5 and 6, respectively.

## Literature survey

Deep learning has been successfully applied in many areas of medical imaging like diabetic retinopathy, histological analysis, cardiac imaging, tumour detection, etc. Consequently, deep learning-based approaches have also been applied to detect COVID-19 using radiological images of chest X-Rays, CT scans, etc.

In the work by [26], the authors have proposed an adaptive feature selection guided deep forest (AFS-DF) for the purpose of COVID-19 detection using the chest CT images. They have first extracted location-specific features from the images and have then applied a deep forest model in order to capture the high-level representation of these features with such small-scale data. They have also proposed a feature selection method for the deep forest model for reducing the redundancy of features. The feature selection method has been adaptively incorporated with the COVID-19 classification model. The authors have evaluated their model on the COVID-19 CT scan dataset with 1495 patients of COVID-19 and 1027 patients of community acquired pneumonia (CAP). Their method has achieved 91.79% accuracy, 93.05% sensitivity, 89.95% specificity and 96.35% AUC, respectively.

Singh et al. [25], in their work, have used a convolutional neural network (CNN) to classify the COVID-19 patients as infected or not. A noticeable point of this work is that the initial parameters of the CNN have been tuned using multi-objective differential evolution (MODE). The authors have performed extensive experiments by considering their approach and the state-of-the-art machine learning techniques on the chest CT images. The authors have reported that their proposed model can classify the chest CT images at a good accuracy rate of over 90%.

The availability of a large and varied sample of data (X-Rays, CT scans, etc.) is important for the generalizability and predictive power of deep learning based models. However, in reality, there is often a lack of suitable data in some domains. The work by [30] aims to tackle this problem by producing synthetic data of normal and COVID-19-affected chest X-Rays using a generative model. They have developed a modified auxiliary classifier generative adversarial network (ACGAN) which they term as CovidGAN for the generation of synthetic images. The authors have observed that the inclusion of the synthetic data in a VGG16 classifier leads to a marked improvement in the performance metrics. The accuracy and F1 score increase to 95% and 0.94 from 85% and 0.84, respectively.

Several works also apply transfer learning and ensembling approach to improve the performance of the models as opposed to training a model from scratch.

The authors in the work by [14] have applied transfer learning using several models which were previously trained on the ImageNet dataset. They have noted that the DenseNet201 model performs the best, as compared to VGG16, ResNet152V2 and InceptionResNetV2. They have used the SARS-CoV-2 CT scan dataset^1^ from Kaggle for evaluating their approach. The dataset consists of 2492 CT scans out of which 1262 are COVID-19 positive and the remaining are COVID-19 negative. The authors have reported training, validation and testing accuracies of 99.82%, 97.40% and 96.25%, respectively.

Similarly, the work by [22] combines ensembling with iterative pruning to detect pneumonia-related and COVID-19-related abnormalities from chest X-Rays. The authors use a combination of various datasets as is mentioned in their work. One notable stage in their pipeline is the modality-specific training. The models are pretrained on a pneumonia-related chest X-Ray dataset to learn task-specific feature representations. The reasoning is that since COVID-19-related data (chest X-Rays) is limited, the pretraining can help the models to generalize better. The authors report their accuracy and AUC as 99.01% and 0.9972, respectively.

In the work by [19], the authors propose a novel 3D convolutional network with an online attention module for detecting COVID-19 in chest CT images. They perform their training and validation on a multi-centre CT data from eight hospitals comprising of 2186 CT scans from 1588 patients. For the testing stage, a similar dataset of 2796 CT scans from 2057 patients was used. A custom 3D ResNet34 architecture with an attention module is proposed by the authors. Two models are trained, one with uniform sampling from the training data and the other with size-balanced sampling due to class imbalance in the data. The predictions from the two models are then combined using an ensemble learning strategy. The authors report the AUC, accuracy, sensitivity, specificity and F1 score as 0.944, 87.5%, 86.9%, 90.1% and 82.0%, respectively.

Goel et al. [8] propose an optimized convolutional neural network (OptCoNet) for the automatic diagnosis of Covid-19 from chest X-Rays. The proposed architecture is composed of feature extraction and classification components like a CNN. However, the hyperparameters of the CNN have been optimized using the grey wolf optimizer (GWO) algorithm. The data comprised of chest X-rays of normal and pneumonia-affected patients collected from publicly available repositories. There were 2700 images in all, of which 900 were Covid-19 images. The authors reported that the optimized CNN model outperforms the state-of-the-art models. The reported accuracy, sensitivity, specificity, precision and F1-score values are 97.78%, 97.75%, 96.25%, 92.88% and 95.25%, respectively.

The idea of mimicking brain cells is not a new domain. In fact neural networks themselves are a representation of neurons present in a human brain. But with the increasing energy demands and depleting energy resources followed by the advent of neuromorphic computing various new works have started evolving in the domain.

In the paper by [13], the authors have proposed a simple approach of spike response model (SRM) neuron with high computational efficiency. For representing data, they have used frequency spike coding based on receptive fields and encoded the images by the network. The method they have used for processing the images is equivalent to the mechanism followed by the primary layers in visual cortex. The output of the network has then been used for extracting primary features for refined classification. The authors have reported that the model has successfully learnt and classified greyscale images with added noise or partially ambiguous image samples at a 20x higher speed at an equivalent classification ratio as compared to a classic SRM neuron membrane model. Their solution is a combination of network topology, spike encoding and neuron membrane model.

### Research gap

Deep learning-based approaches have become very popular in the recent years. It has been applied in several domains of image processing, pattern recognition and computer vision to achieve state-of-the-art results. In particular, the fields of medical image processing have seen many advances recently in various tasks that include image classification, image segmentation, image retrieval, computer-aided diagnosis, etc. Some works relating to the domain of COVID-19 have already been mentioned above. On the other hand, the applications of SNNs are a relatively new topic in research. As theoretical research is still ongoing, very few application-based works have been published. To the best of our knowledge, no SNN-based works have been proposed till date in the field of COVID-19 detection from chest CT scans or CXRs. Therefore, our work might be the first such work.

## Dataset used

**Fig. 1:**
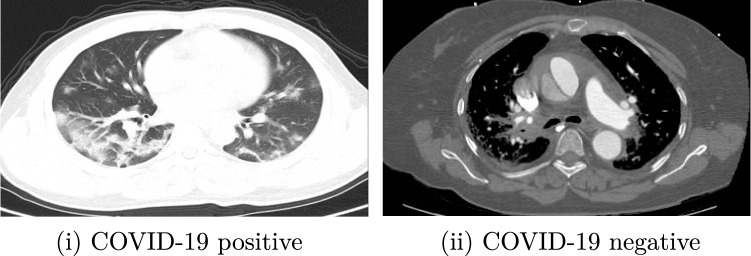
Sample images from COVID-19 CT Scan dataset belonging to corresponding classes

The COVID-CT dataset^2^ contains 349 CT scan images of 216 patients (multiple images for same patient taken at different times) diagnosed positive for COVID-19 and 397 CT scan images that are diagnosed negative for COVID-19. The dataset is open-sourced to the public, to foster the research works of CT specific testing of COVID-19. From 760 medRxiv and bioRxiv preprints about COVID-19, the creators of the dataset extracted reported CT images and manually selected those containing clinical findings of COVID-19 by reading the captions of the selected images. The credibility and effectiveness of the dataset have been confirmed by a highly qualified senior radiologist who has intensively diagnosed and treated many COVID-19 patients. The personal data of the patients are anonymized to protect their privacy [28]; there is a metadata file that allows access to the data through descriptors such as patient ID, patient information, DOI and image caption. Some sample images from the dataset are shown in Fig. 1.

## Proposed methodology

**Fig. 2:**
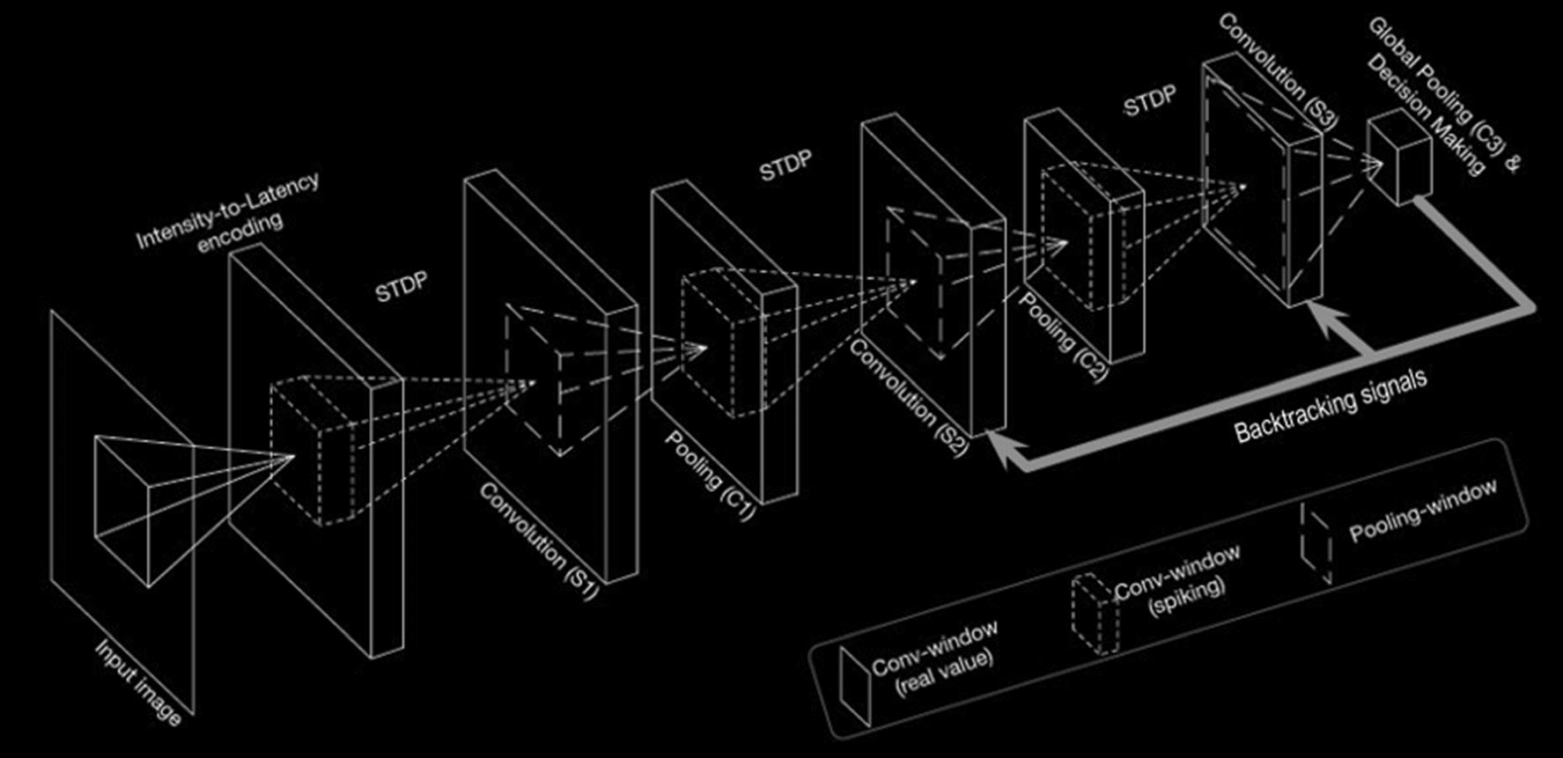
Overall architecture used for feature extraction

In this section, the working principle of the proposed model has been described. The overall methodology is shown in Fig. 2.

### Overall architecture

The work of the input layer is to encode the input image in the form of a Poisson-distributed spike. The pixel intensity has a directly proportional relationship with the probability of spike generation. This resultant encoding is passed on to the intermediate stages. The intermediate stages of feature hierarchies consist of hidden layers which are made up of convolution and spatial-pooling (C) layers stacked alternatively. These spikes are then concatenated sequentially and used as features to be fed to external classifiers for binary classification of the inputs. Apart from the C layers, all the other layers consist of trainable parameters. The local features which are having spatial correlation in the input patterns can be detected by the adapted convolutional kernels using convolution, which has an intrinsic property of rendering the network showing invariance to translation (shift) in the object location. Thereafter, downscaling of feature maps in terms of dimension which are produced by the previous layers is done by the P layer. In the whole process, retention of the spatial correlation between neighbourhood pixels in every feature map takes place.

### Preprocessing

**Fig. 3:**
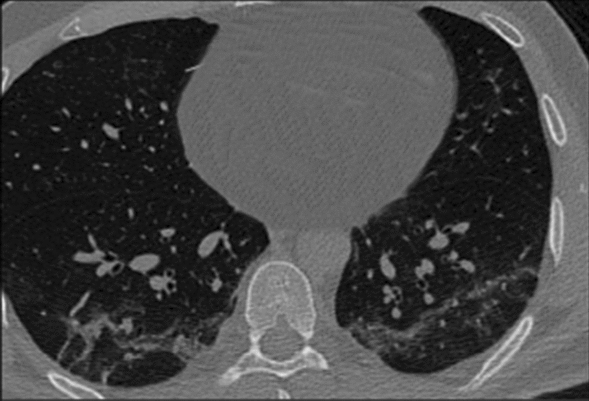
Sample image before preprocessing

**Fig. 4:**
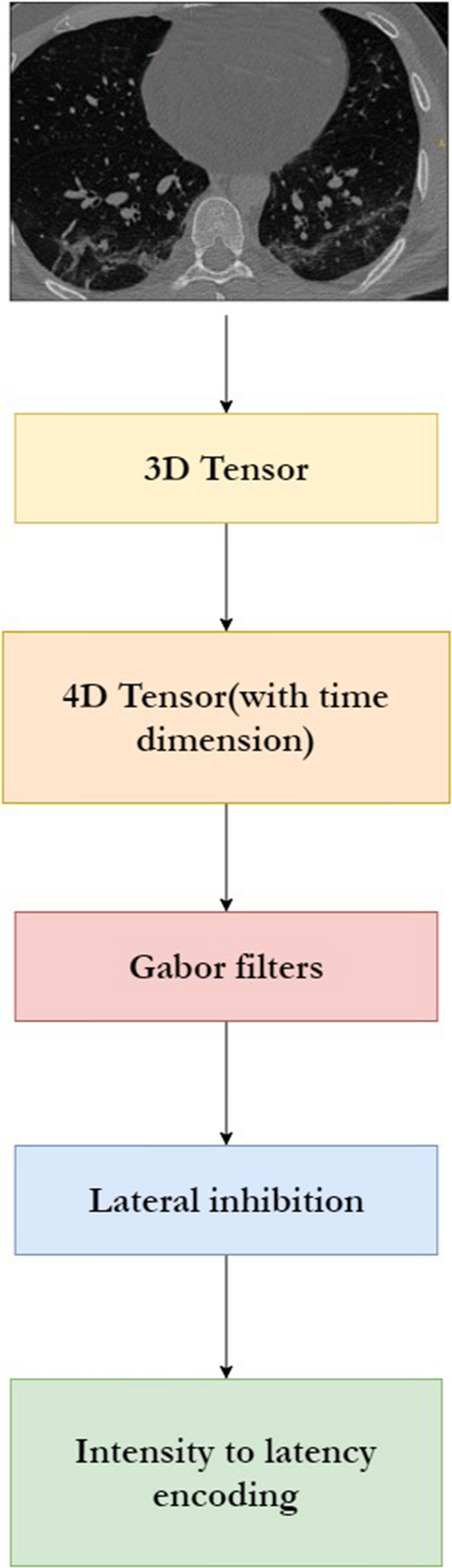
Image preprocessing pipeline

Every image is first resized to a dimension of $32\times 32$ and converted to greyscale. This image (as shown in Fig. 3) is then passed through the preprocessing pipeline as shown in Fig. 4 and the resultant encoded image is passed through the network for further processing.

#### **Gabor filters**

Gabor filters are a type of band-pass filters which prove to be quite useful in extracting useful features from images. Multiplication of a Gaussian envelope function with a complex oscillating function generates the impulsive response of these filters. [5] had shown in his work that these simple functions help in minimizing the space-time uncertainty product with an immediate implication of orientation selective behaviour of these functions on extension to two dimensions. We have used the filters for extracting various features from the images, both frequency based as well as orientation based features, which have helped the SNNs in better understanding and setting of membrane potentials and spiking thresholds, while filtering out any noise. From these images two features, namely, Gabor filter entropy and Gabor filter energy, are extracted using the real and imaginary components. These features correctly respond to the edges if the edge direction is perpendicular to the Gaussian kernel wave vector.

#### Output of preprocessing stage

**Fig. 5:**
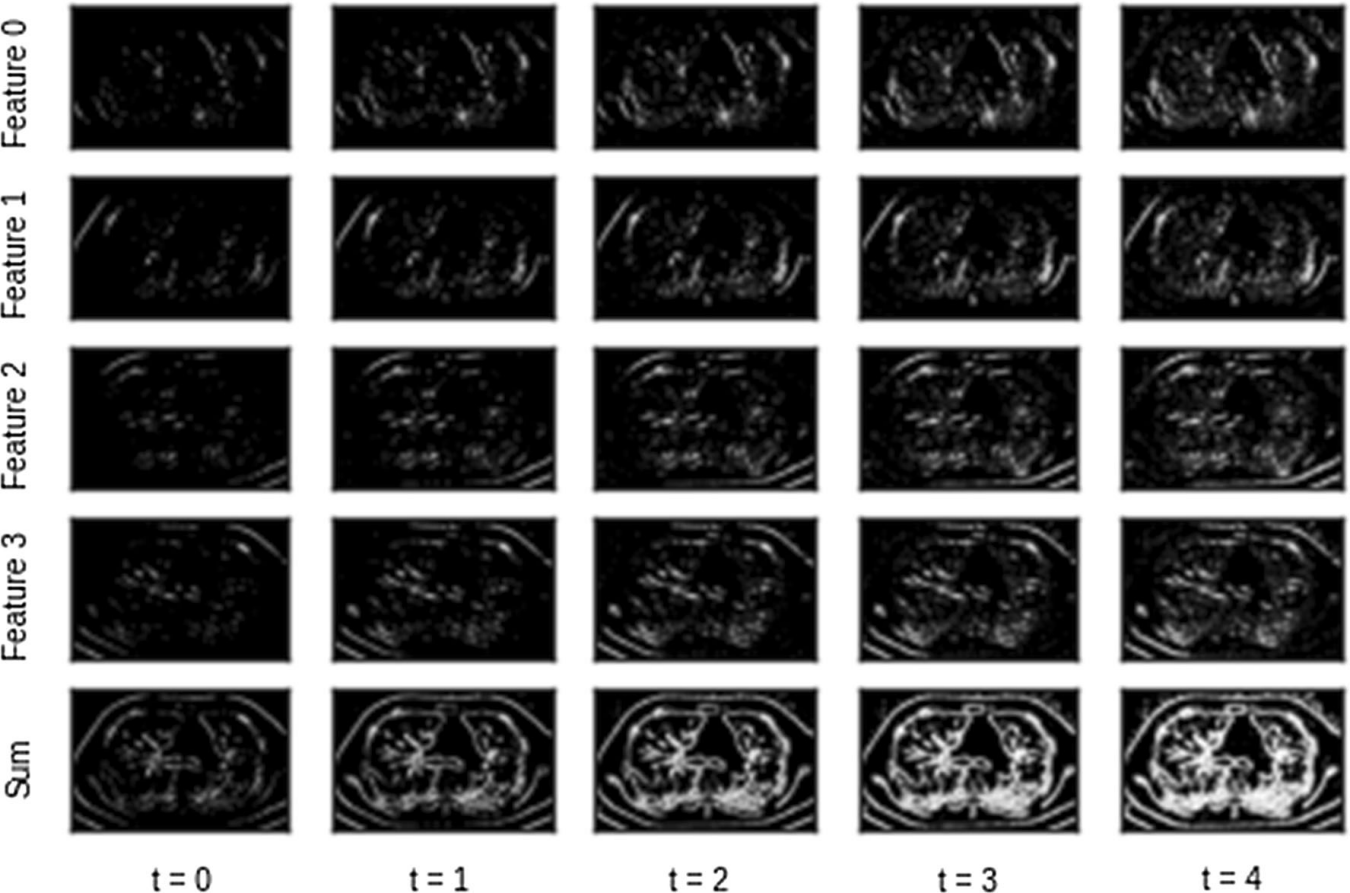
Visualization of features after preprocessing

Figure 5 provides a visualization of the features after the preprocessing stage for a single input image. The time values in the bottom are a result of adding the time dimension. The different features are a result of applying the different Gabor filters. The summation of these features produces the final feature vector. The features and hence their summation are different at different time intervals. This is due to the intensity-to-latency encoding which is applied in the last stage of preprocessing.

### Feature extraction

Here we have explained in a detailed manner the feature extraction process of the architecture.

#### Convolution

Every convolutional (S-layers) in the proposed network consists of many 2D grids of Integrate-and-Fire (IF) neurons, which is basically the representation of feature maps. Every neuron belonging to a layer has a definite three-dimensional input window with same height and width of afferent which is analogous to the units conveying information from sensory organs to central nervous system and depth which is same as the number of feature maps present in the previous layer [17]. The firing threshold is also initialized to be equal across all the neurons in every individual layer. In every time step, the value of internal potential for every IF neuron is enhanced by the incoming spikes within its input window by making use of the magnitude of the synaptic weights. There is absence of any leakage in these neurons. If any neuron attains the firing threshold, it emits a single spike, and thereafter, it remains silent until the next input image is passed through the network.

Whenever the network encounters a new input, the internal potentials of all neurons are reset to zero. In a single feature map, a weight-sharing mechanism is applied to all the neurons. A convolutional layer with a kernel size $\mathbb B_h \times \mathbb B_w$ performs a convolution over an input 4D tensor depicting a spike-wave of size $T_{max}\times \mathbb F_{in} \times \mathbb H_{in} \times \mathbb S_{in}$ having value of stride equal to 1 and produces a tensor of output potentials having size $T_{max}\times \mathbb F_{out} \times \mathbb H_{out} \times \mathbb S_{out}$, where:

1 $$\begin{aligned} \mathbb H_{out} = \mathbb H_{in} - \mathbb B_h + 1, \mathbb S_{out} = \mathbb S_{in} - \mathbb B_h + 1 \end{aligned}$$

where $\mathbb F_{in}$ and $\mathbb F_{out}$ represent the values corresponding to number of input and output features, respectively. Potential type tensors $(\mathbb P)$ are similar to the binary spike-wave tensors, but $\mathbb P[t, f, r, c]$ represents the floating-point potential of any neuron placed at the position (*r*, *c*) of any feature map *f*, at time step *t* (see Table 1).

**Table 1:** Parameter configuration for S-layers for feature extraction [18]

Layer	Number of feature maps	Input window (width, height, depth)	Threshold
S1	30	(5, 5, 6)	15
S2	250	(3, 3, 30)	10
S3	200	(5, 5, 250)	$\infty$

#### Spike timing-dependent plasticity

**Fig. 6:**
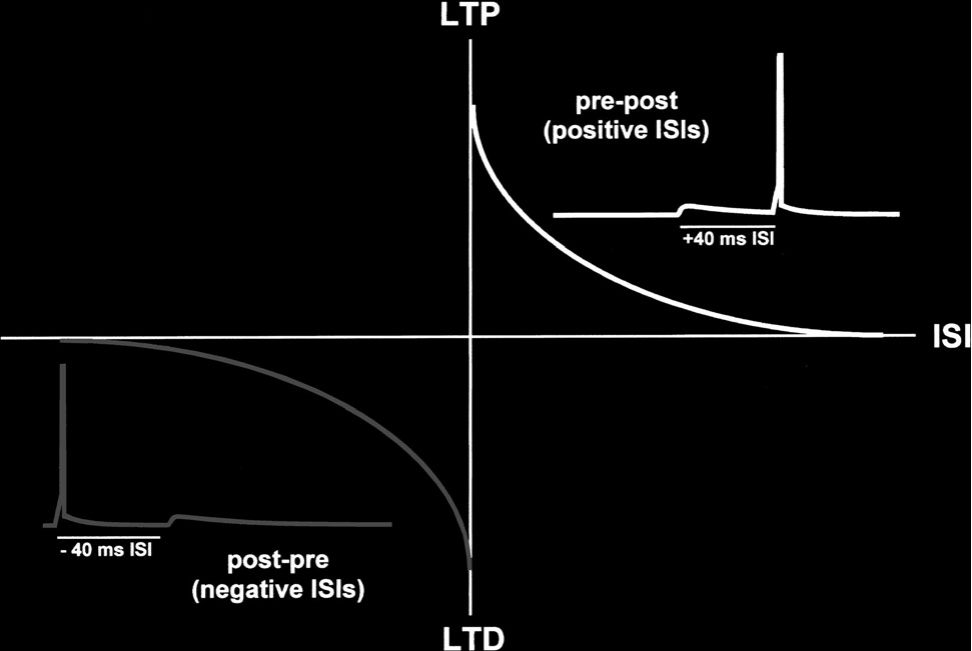
Presynaptic and post-synaptic spike pairs

Spike timing-dependant plasticity (STDP) is a proven technique for detecting hidden patterns from the noise present in spiking data. It belongs to the class of unsupervised learning techniques which works on the basis of the ordering of the synaptic spikes. The ordering of every pair of presynaptic spike and post-synaptic spikes as shown in Fig. 6 decides the potentiation (pre-post) or depression (post-pre) of the synapse. Changes of weights is based on the following two rules:

- Any synapse that contributes to the firing of a post-synaptic neuron should be made strong, that is, its value should be increased.
- Synapses that do not contribute to the firing of a post-synaptic neuron should be diminished, that is, its value should be decreased.

This method helps in learning the repetition of patterns among a large set of incoming spikes.

Since this simulation of SNN works with the coding mechanism of time to first spike, the implemented STDP function that has been used here is shown in Eq. 2:

2 $$ \Delta {\mathbb{S}}_{{i,j}}  = \left\{ {\begin{array}{*{20}l}    {K^{ + }  \times ({\mathbb{S}}_{{i,j}}  - L) \times (U - {\mathbb{S}}_{{i,j}} ){\text{ if }}T_{j}  - T_{i} } \hfill & { \le 0,} \hfill  \\    {K^{ - }  \times ({\mathbb{S}}_{{i,j}}  - L) \times (U - {\mathbb{S}}_{{i,j}} ){\text{ if }}T_{j}  - T_{i} } \hfill & { > 0} \hfill  \\   \end{array} } \right.{\text{ }} $$

where $\mathbb S_{i,j}$ refers to the corresponding change of value of weights of the synapse which connects the post-synaptic neuron i to the presynaptic neuron j, $K^+$ and $K^-$ signify the learning rates, and $(\mathbb S_{i,j}-L)\times (U-\mathbb S_{i,j})$ is the regularizer slowing down the change in weight when the synaptic weight $(\mathbb S_{i,j})$ is nearer to the lower(L) and upper(U) bounds. $T_i$ and $T_j$ depict the spike times of the presynaptic (input) and the post-synaptic (output) neuron, respectively (see Table 2).

**Table 2:** Parameter configuration for synaptic plasticity for feature extraction

Layer	$K^+$	$K^-$	k	r
S1	0.004	−0.003	5	3
S2	0.004	−0.003	8	2
S3	0.004	−0.003	1	0

For applying STDP in the process of training, provision of the input and output spike waves, as well as output potentials, are necessary in order to find the winners. Winners-take-all (WTA) is a well-known competition-based algorithm which is employed in SNNs. WTA is generally used for plasticity, but is extensible for use for performing other functions such as decision-making. Winners are decided first on basis of the earliest spike times, thereafter based on the maximum potentials. The number of winners is defaulted to take the value 1. The phenomenon of lateral inhibition is followed by means of which the winners’ surrounding neurons present in all of the feature maps within a specific distance are completely inhibited. This helps in enhancing probability of learning more diverse features.

**Fig. 7:**
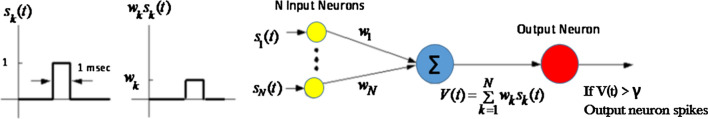
A simple two-layer network with presynaptic($s_i$) and post-synaptic(output) neurons

Figure 7 [27] represents a simple two-layer network consisting of N presynaptic neurons (input) and 1 output neuron. The signals of spikes ($s_i(t)$) are designed to be either 0 or 1 in one millisecond of increment. That is, 1 millisecond pulse of amplitude 1 depicts a spike and a value of 0 depicts absence of a spike. Every signal of spike contains a weight or synapse associated with it which gets multiplied with the signal to obtain $w_is_i(t)$ which is termed as the post-synaptic potential due to the $i^{th}$ input neuron. These potentials are thereafter aggregated using the Eq. 3.

3 $$\begin{aligned} \mathcal {V}(t)=\sum _{k=1}^N w_ks_k(t) \end{aligned}$$

where, $\mathcal {V}(t)$ is termed as the membrane potential of the output neuron. At some instant of time t, if the membrane potential $\mathcal {V}(t)$ exceeds a specified threshold, that is, if $\mathcal {V}(t) > \gamma$, then there is a spike in the output neuron.

#### Pooling

Pooling layers (C-layers) have been used for the purpose of position invariance and reduction of information redundancy. Every C-layer or S-layer consists of equal number of feature maps as present in its previous layer, thus building a one-to-one relationship between the maps of both layers. For two variants of SNNs, which are spike train-based and potential-based, we have used two types of pooling layers, respectively. Both the variants consist of a 2D input window and a fixed stride. Every neuron in the layers gives indication of the maximum potential and the earliest spike time of the neurons within its input window for the potential based and spike train-based layers respectively. The value of stride is same as the window size by default, but can be customized accordingly.

**Fig. 8:**
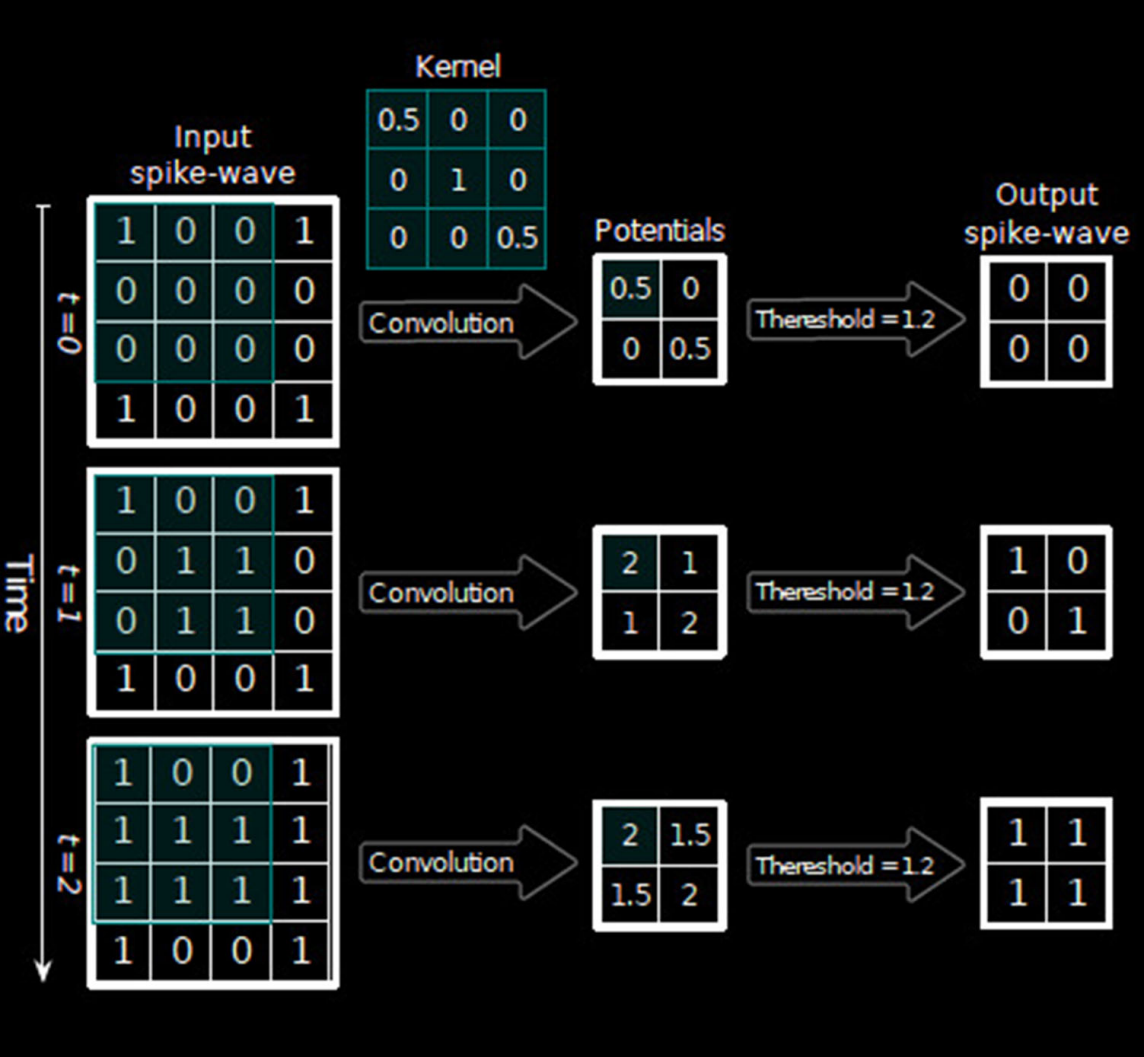
Simultaneous processing of spikes over time steps to convert to tensor containing potentials in all of the time steps

According to the structure of the spike-wave and potential tensors, if a spike-wave tensor serves as the input, then the earliest spike within each pooling window is extracted, whereas if a potential-based tensor serves as the input, the maximum potential within each pooling window is extracted. The feature maps of the final pooling layer are flattened to give a one dimensional vector which is fed to an external classifier whose work in turn is to produce inference decisions. The classifier effectively incorporates the composition of features which are the results from the alternating convolutional and pooling layers and classifies into the final output classes. Figure 8 shows an example of conversion of spike times into tensors of spike-wave (see Table 3).

**Table 3:** Parameter configuration for C-layers for feature extraction [18]

Layer	Input window (width,height)	Stride	Type
C1	(2,2)	2	Spike train-based
C2	(3,3)	3	Spike train-based
C3	(5,5)	0	Potential based

### Classifier

**Fig. 9:**
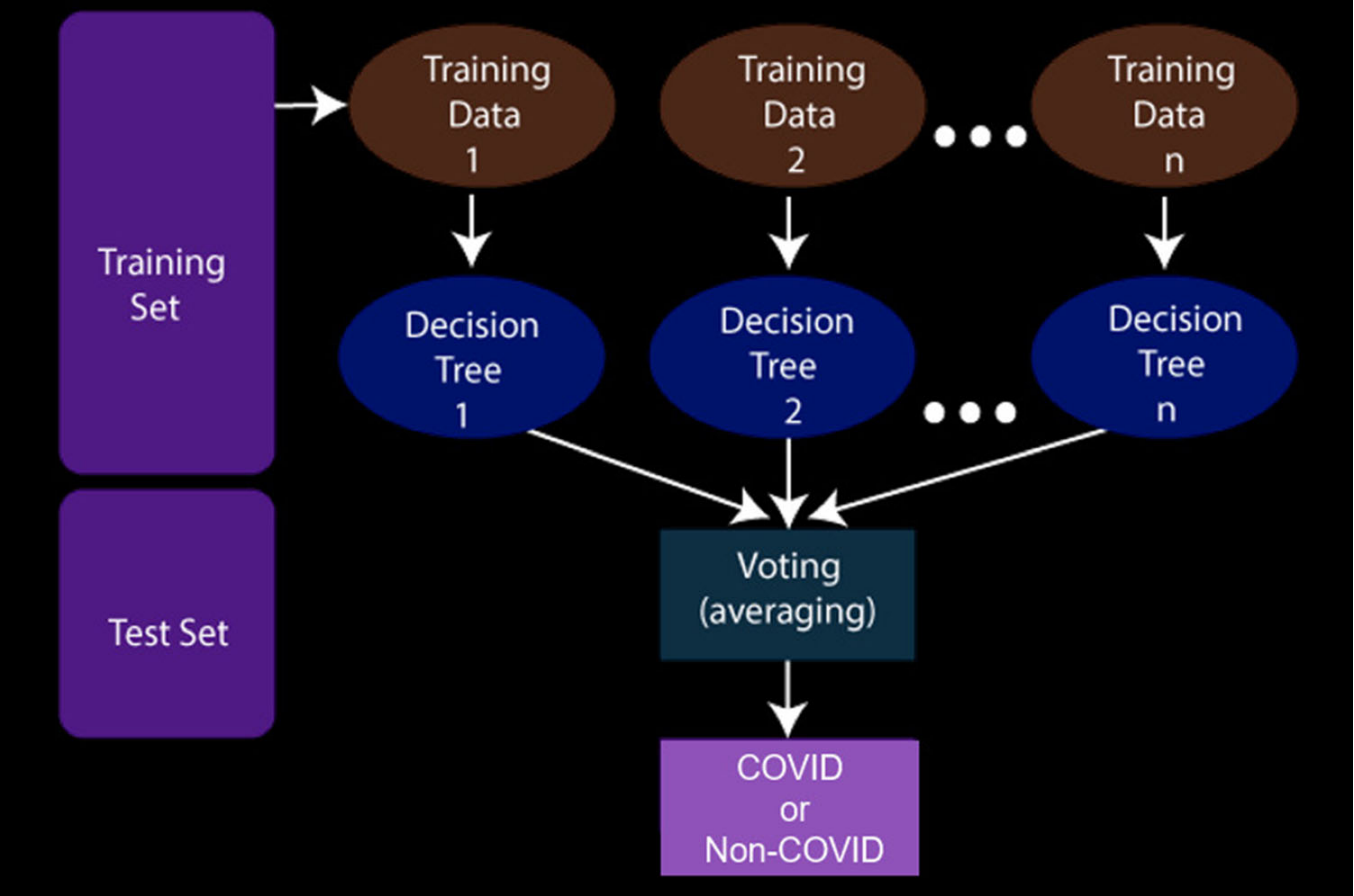
Overview of working of a random forest classifier

**Fig. 10:**
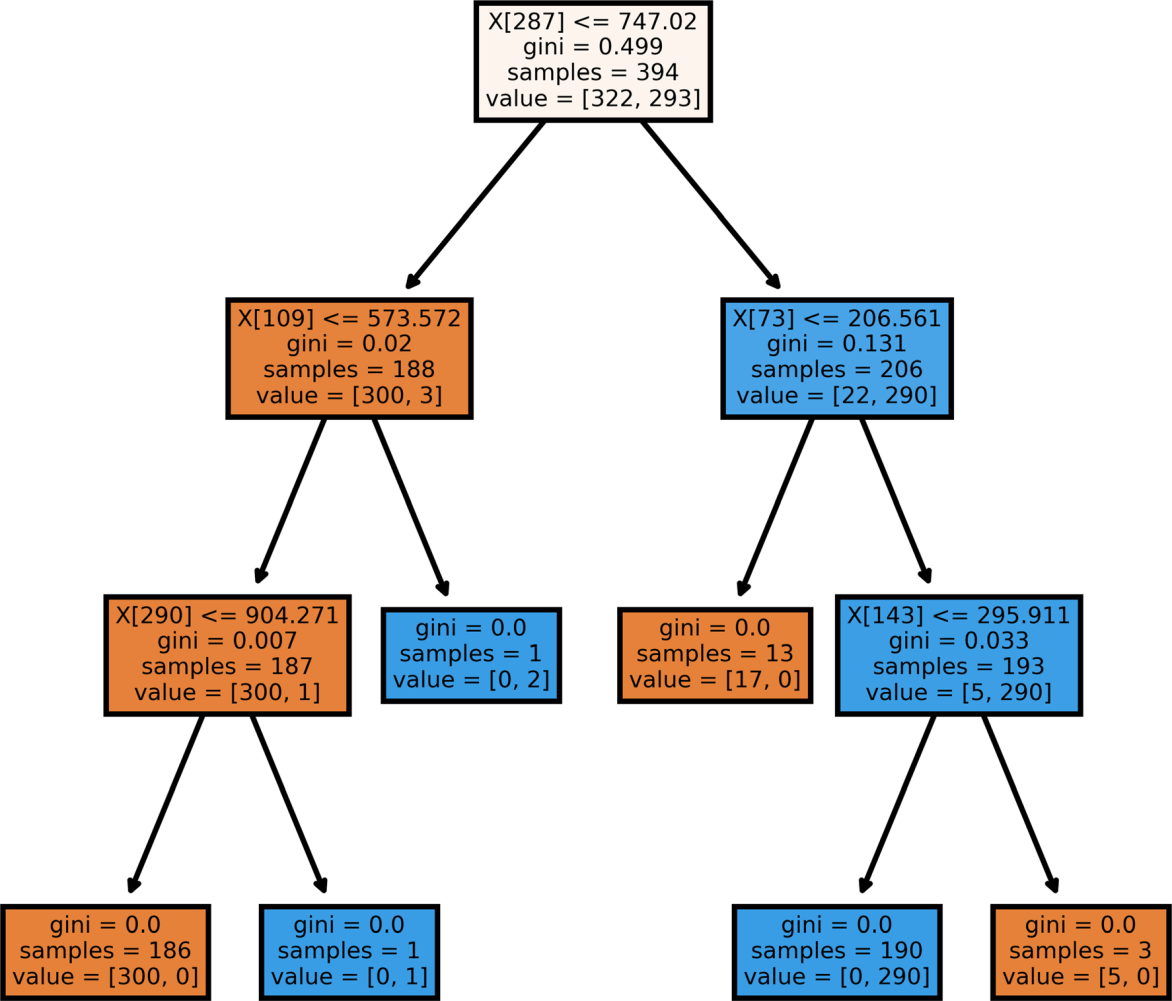
Sample decision tree of an estimator of the random forest classifier

Deep learning-based classifiers are the best when there is presence of a huge number of training instances. However, if a dataset contains lesser quantities of data, machine learning-based classifiers are best suited for the decision making stage [29]. In this work, at the last level of the whole working pipeline, we have used a Random Forest classifier [2] for classifying the output class of COVID or non-COVID on the basis of the features extracted in the upper levels of the working pipeline. A random forest classifier is an ensemble-based classifier that generates a series of decision tree classifiers (shown in Fig. 10) on various sub-samples of the dataset as shown in Fig. 9. It uses the principle of averaging to improve the accuracy of prediction and to control over-fitting. For our work, we have set the maximum depth hyper-parameter to 8, random-state to 32 and other parameters to their default values as initialized in the scikit-learn library [21].

### Comparison with deep learning models

We also train some state-of-the-art deep learning-based classifiers to find the performance of these models on the metrics considered. We use these as baselines in order to judge the performance of the proposed approach against the prevalent deep learning based approaches. The models considered are: VGG16, ResNetV2 and Densenet161 adapted from the works by [, 10, 11, 24] respectively.

**Fig. 11:**
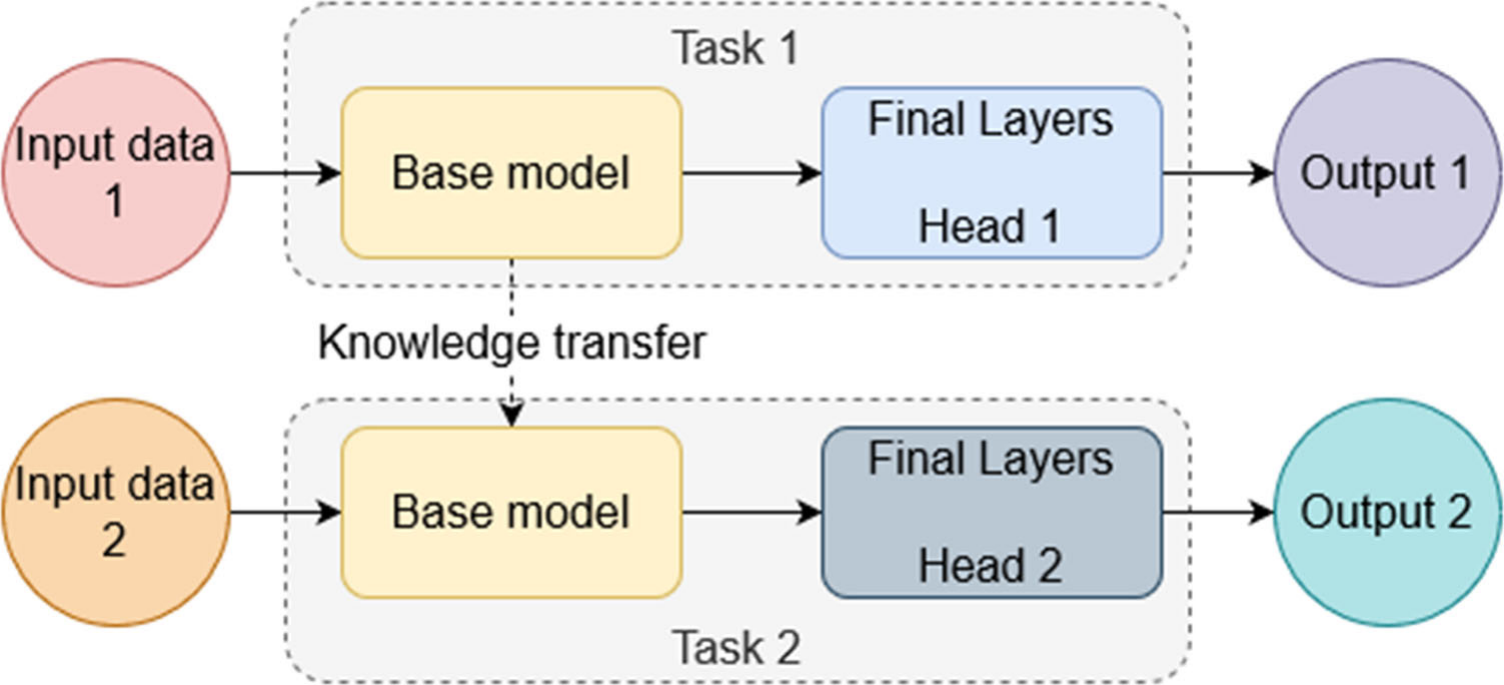
A schematic diagram of the transfer learning approach

We use the transfer learning technique to train the models under consideration. A schematic diagram of the technique is highlighted in Fig. 11. Initially, the models are trained on the ImageNet dataset^3^. After convergence, the last dense layer (Head 1 in the figure) is replaced with a randomly initialized dense layer (Head 2 in the figure) with two outputs corresponding to the two classes. The model is then trained for 20 epochs with only the weights of the last layer being updated. After that, the entire model is fine-tuned for 20 epochs with a very low learning rate.

## Experimental results

### Metrics used

We have used various classification metrics [16] for measuring the effectiveness of our model in classifying COVID from the CT scan images, which are as follows:

- F1-score: Weighted average of precision and recall values.
- Accuracy: Overall probability of a patient being correctly classified.
- Sensitivity: Probability of positive test results when person is actually having the disease .
- Specificity: Probability of negative test results when person is actually not having the disease.
- Positive likelihood ratio: Ratios of the probabilities of a test result being positive provided the disease is present and a test result being positive given the disease is absent.
- Negative likelihood ratio: Ratios of the probabilities of a test result being negative provided the disease is present and a test result being negative given the disease is absent.
- Positive predictive value: Probability that provided the test is positive, the disease is present.
- Negative predictive value: Probability that provided the test is negative, the disease is absent.

### Results and analysis

At the outset, it is to be noted that training the deep learning models from scratch results in overfitting on the training dataset. This leads to poor performance on the test dataset, with the accuracy being close to approximately 60% while the training accuracy is 100%.

The potential-based classification achieves much better results compared to the spike train-based model as shown in Tables 4, 5, 6 and 7 . This occurs due to the collective errors which keeps on accumulating due to improper firing of spikes resulting due to improper thresholding at the IF neurons while using spike train-based classification. This error accumulation is absent in case of potential based model due to direct relationship of the potential of neurons with the decision making. This can be understood from the natural phenomenon itself which the SNNs mimic.

Action potential marks the beginning of the chain of events which lead to contraction for example in muscle cells, while the temporal sequence of these action potentials are called spike trains. So errors occurring in the action potentials propagate and keep on accumulating in the spike trains.

**Table 4:** Classification results of potential-based model

Class	Precision	Recall	F1-score	Support
COVID-19	1.00	0.99	0.99	95
Non-COVID	0.99	1.00	0.99	110
**Macro avg.**	1.00	0.99	0.99	205

**Table 5:** Medical results [23] associated with the classification using potential-based model

Statistic	Value	95% CI
Sensitivity	98.96%	94.33% to 99.97%
Specificity	100.00%	96.67% to 100.00%
Negative likelihood ratio	0.01	0.00 to 0.07
Disease prevalence (*)	46.83%	39.84% to 53.91%
Positive predictive value (*)	100.00%	
Negative predictive value (*)	99.09%	93.94% to 99.87%
Accuracy (*)	99.51%	97.31% to 99.99%

**Table 6:** Classification results of spike train-based model

Class	Precision	Recall	F1-score	Support
COVID-19	0.63	0.92	0.74	95
Non-COVID	0.88	0.54	0.66	110
**Macro avg.**	0.75	0.72	0.70	205

**Table 7:** Medical results associated with the classification using spike train-based model

Statistic	Value	95% CI
Sensitivity	91.58%	84.08% to 96.29%
Specificity	53.64%	43.88% to 63.20%
Positive likelihood ratio	1.98	1.60 to 2.44
Negative likelihood ratio	0.16	0.08 to 0.31
Disease prevalence (*)	46.34%	39.37% to 53.42%
Positive predictive value (*)	63.04%	58.03% to 67.79%
Negative predictive value (*)	88.06%	78.79% to 93.61%
Accuracy (*)	71.22%	64.50% to 77.31%

**Fig. 12:**
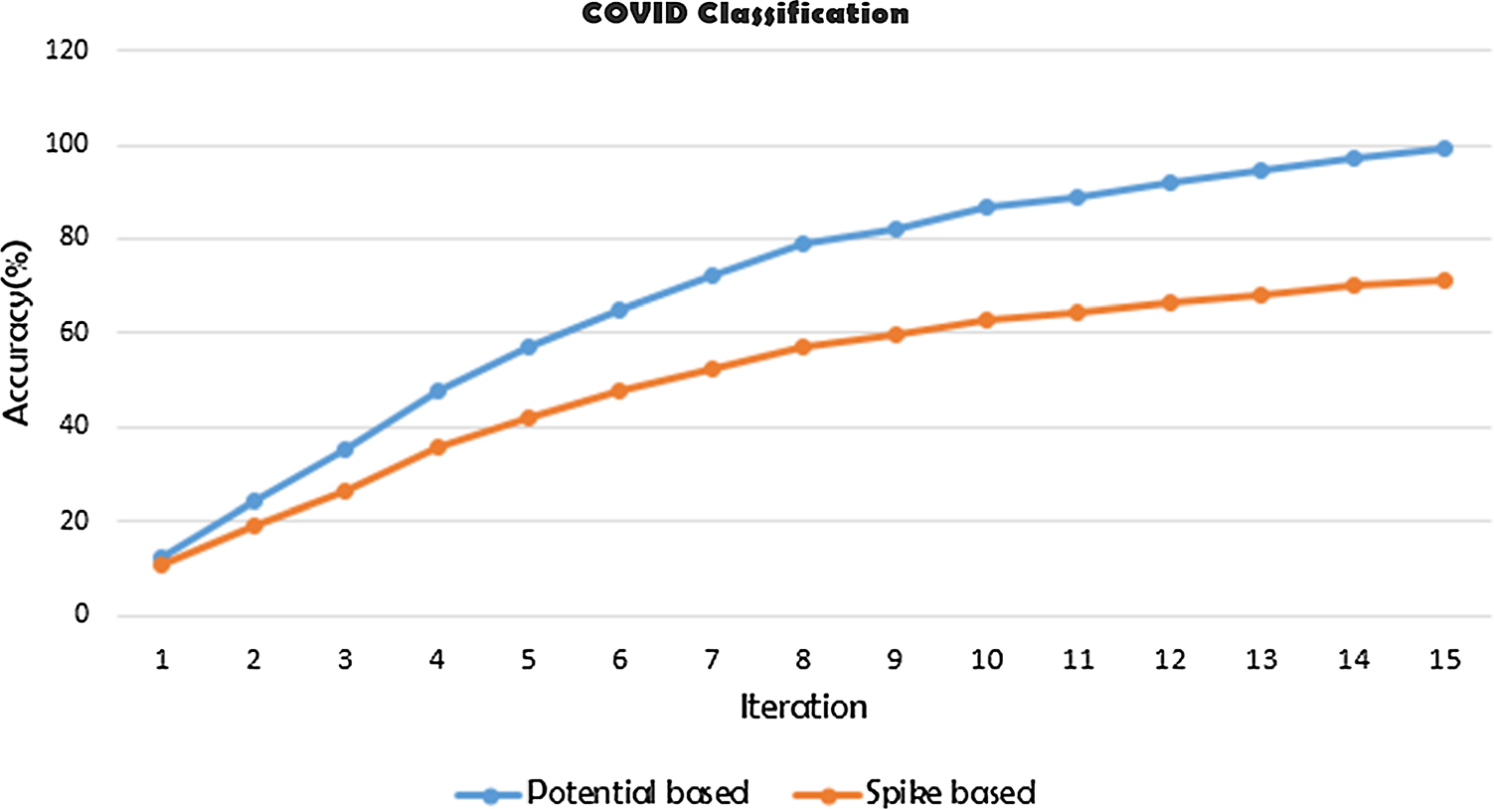
Iterative accuracy attained while training in potential and spike train-based feature classifiers

The learning in the network goes on quite smoothly as it can be seen from the iterative training accuracy graphs in Fig. 12. Both the potential-based and spike train-based models start to learn with almost equal accuracy but with time the learning of the spike train-based model does not keep up with the learning capabilities of the potential-based model and hence gives worse results comparatively because of exactly the same reason as explained above.

Table 8 shows the precision, recall and F1-score of the VGG [24], ResNet [9] and DenseNet [12] models, respectively. The models perform similarly, with the VGG having a F1 score about 0.02 lower than the other two models both of which achieve scores of 0.88. On comparison with the spike-based model (see Table 6), we find that the deep learning models have better performance by a large margin. However, with respect to the potential based model (see Table 4), we find that these models are lacking in their performance based on the metrics being considered.

**Table 8:** Classification results of the deep learning-based models

Model	Class	Precision	Recall	F1-score	Support
VGG16	COVID	0.83	0.88	0.85	86
	Non-COVID	0.89	0.84	0.87	101
	Macro avg.	0.86	0.86	0.86	187
ResNet101V2	COVID	0.83	0.92	0.87	86
	Non-COVID	0.92	0.84	0.88	101
	Macro avg.	0.88	0.88	0.88	187
DenseNet161	COVID	0.89	0.85	0.87	86
	Non-COVID	0.88	0.91	0.89	101
	Macro avg.	0.88	0.88	0.88	187

Table 9 highlights the accuracies of all the models that have been considered in this work. The potential-based model outperforms all the other models considered here. The errors in the spike and potential based models are highlighted in Table 10. It can be noted that the potential-based model performs better than the deep learning models. It can be because the potential-based model learns fewer redundant features with respect to the other convolution-based deep learning models. Having fewer parameters as compared to the deep learning models, it also makes more efficient use of the parameters. However, one trade-off for this higher performance and efficiency would be the higher training time for the SNN based models.

**Table 9:** Accuracy of the models considered

Model	Accuracy
VGG16	0.86
ResNet101V2	0.88
DenseNet161	0.88
Spike train-based	0.71
Potential-based	1.00

**Table 10:** Errors of the two SNN variants considered

Model	Error
Spike train-based	0.29269
Potential-based	0.00488

From the above results, it can be seen that the potential-based SNN model performs the best. The spike-train-based SNN does not provide competitive results as compared to the deep learning-based models. However, the potential-based SNN outperforms the state-of-the-art deep learning models by a significant margin. This demonstrates the usability of SNNs in real-world scenarios. Additionally, the benefits provided by the neuromorphic chips make SNNs a viable option for various practical applications. This is relevant since deep learning models generally require a graphics processing unit to obtain fast inference times. The energy-efficiency and computing power of the chips are more suited as processing elements. The advantages do come at a cost. It takes a large amount of time to train them, even more than the deep learning models which themselves take hours, or even days in some cases.

## Conclusion

In this work, we have designed a three-layer DCSNN for screening of the COVID-19 from CT scan images. In doing so, the input image is first convolved and processed with Gabor filters. Then, by means of an intensity-to-latency encoding, a wave of spikes is generated. After propagating through a series of convolutional and pooling layers, with neurons having the ability of firing at most once, the spike wave reaches the penultimate layer. Finally, the useful features are extracted that are then fed to a classifier for making the final decision.

On evaluation on the COVID-CT dataset, the proposed approach has achieved an impressive F1 score of 0.99 for the potential-based model. The approach also outperforms some state-of-the-art COVID-19 classification models. Although the proposed SNN-based model performs very well on chest CT images, there is a limitation of this model. It takes more time to train the model in comparison with the traditionally used deep learning models. However, the present model is more efficient compared to these deep learning models. In future, we plan to come up with an idea which will help us to cut down the training time. Another plan is to apply the model to other forms of COVID-19 datasets like chest X-rays which will prove the robustness of the model.

## Footnotes

1 https://www.kaggle.com/plameneduardo/sarscov2-ctscan-dataset.

2 https://github.com/UCSD-AI4H/COVID-CT.

3 http://www.image-net.org/.
